# Antegrade radial laser ablation for a pancreaticojejunal stricture under peroral pancreatoscopic guidance

**DOI:** 10.1055/a-2723-1445

**Published:** 2025-11-04

**Authors:** Takeshi Ogura, Jun Matsuno, Takafumi Kanadani, Junichi Nakamura, Hiroki Nishikawa

**Affiliations:** 1 Pancreatobiliary Advanced Medical Center, Osaka Medical and Pharmaceutical University Hospital, Osaka, Japan; 2 13010 Endoscopy Center, Osaka Medical and Pharmaceutical University, Osaka, Japan; 3 2nd Department of Internal Medicine, Osaka Medical and Pharmaceutical University, Osaka, Japan

A pancreaticojejunal stricture (PJS) can be treated by enteroscopic guidance, but if the approach by this technique fails, endoscopic ultrasound-guided pancreatic duct drainage (EUS-PD) can be considered. To treat a PJS, endoscopic dilation using various devices, such as a balloon catheter, or a surgical approach may be attempted 1 2 . However, there are several disadvantages, such as recurrence of the PJS in endoscopic dilation or invasiveness for patients in a surgical approach. In contrast, the radial incision and cutting (RIC) method has shown promising results for the treatment of stricture 3 , but an enteroscopic approach should be successfully performed prior to this procedure. While EUS-PD has a high technical success rate, device limitations restrict the performance of antegrade RIC. Technical tips for antegrade radial laser ablation (RLA) for the PJS via the EUS-PD route are described.

A 77-year-old man was admitted to our hospital to treat a PJS. He underwent pancreaticoduodenectomy due to pancreatic cancer 2 years earlier. He developed a hepaticojejunostomy stricture and therefore underwent EUS-guided hepaticogastrostomy and EUS-PD. PJS dilation using a balloon catheter was performed previously, but the PJS was not resolved. Therefore, antegrade RLA was attempted. First, the previously deployed plastic stent was removed after guidewire deployment into the main pancreatic duct. Although contrast medium injection was performed, contrast medium flowing into the intestine through the PJS could not be observed ( Fig. 1 ). After tract dilation using a balloon catheter, pancreatoscope insertion was performed antegradely. Upon pancreatoscopic imaging, the PJS with hyperplasia could be identified ( Fig. 2 ). Then, endoscopic holmium laser ablation was performed in a circumferential manner for the PDS to remove scar tissue ( Fig. 3 ). After this procedure, PJS resolution was obtained ( Fig. 4 ), and the contrast medium flowing into the intestine through the PJS was also seen ( Fig. 5 ). After performing a biopsy for the PJS, a plastic stent was deployed without any adverse events ( Video 1 ), and histology showed no malignancy.

**Fig. 1:**
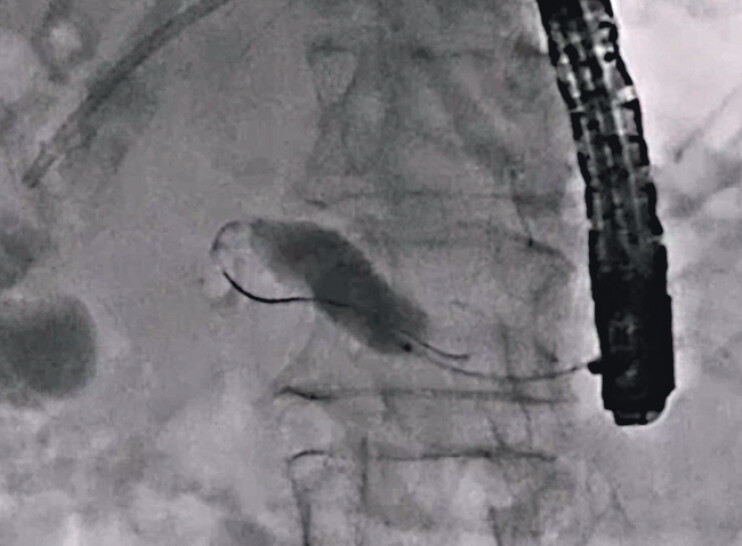
Although contrast medium injection is performed, contrast medium flowing into the intestine through the stricture cannot not be observed.

**Fig. 2:**
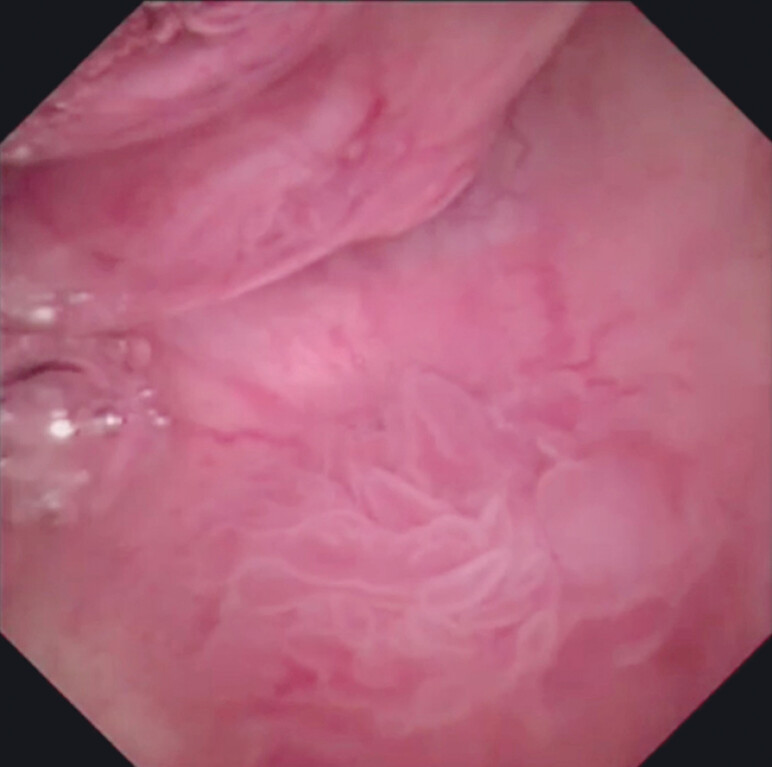
Upon pancreatoscopic imaging, the stricture with hyperplasia can be identified.

**Fig. 3:**
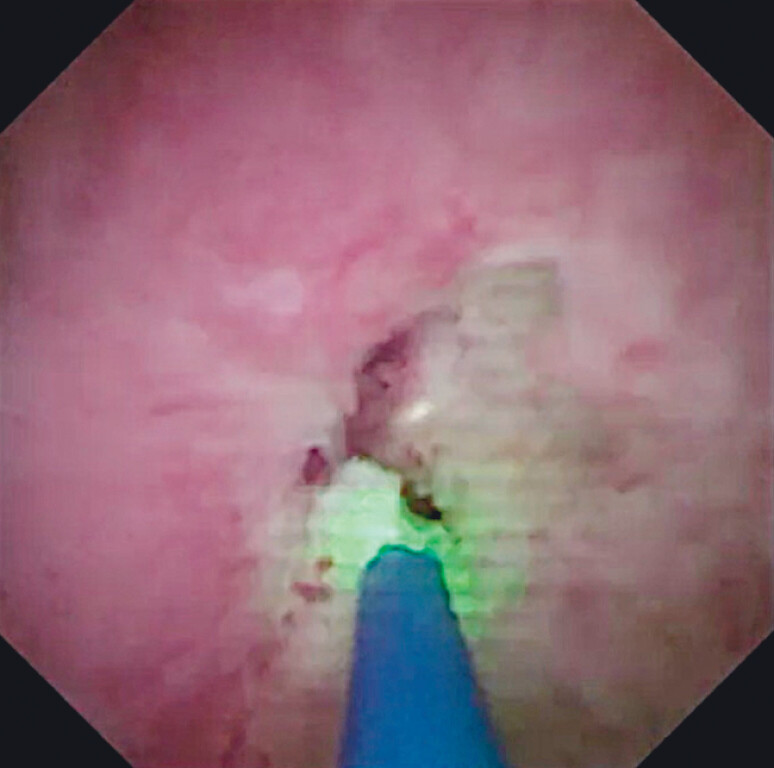
Endoscopic holmium laser ablation is performed in a circumferential manner to remove scar tissue.

**Fig. 4:**
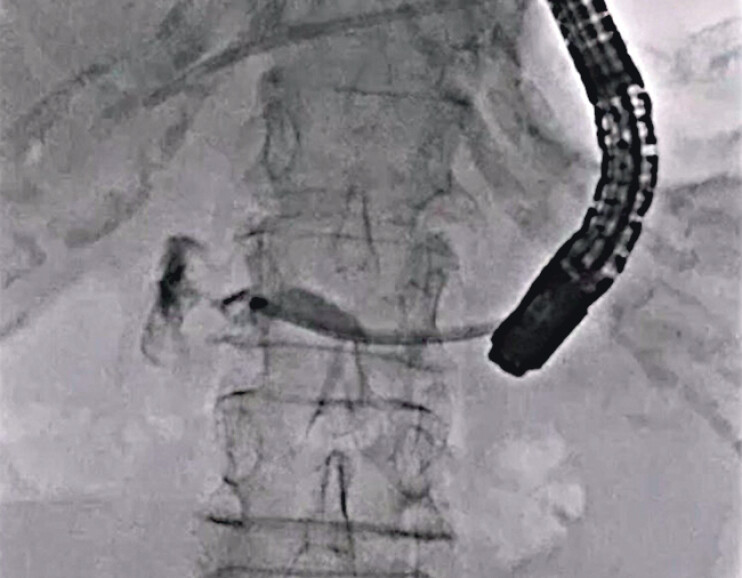
Stricture resolution is obtained.

**Fig. 5:**
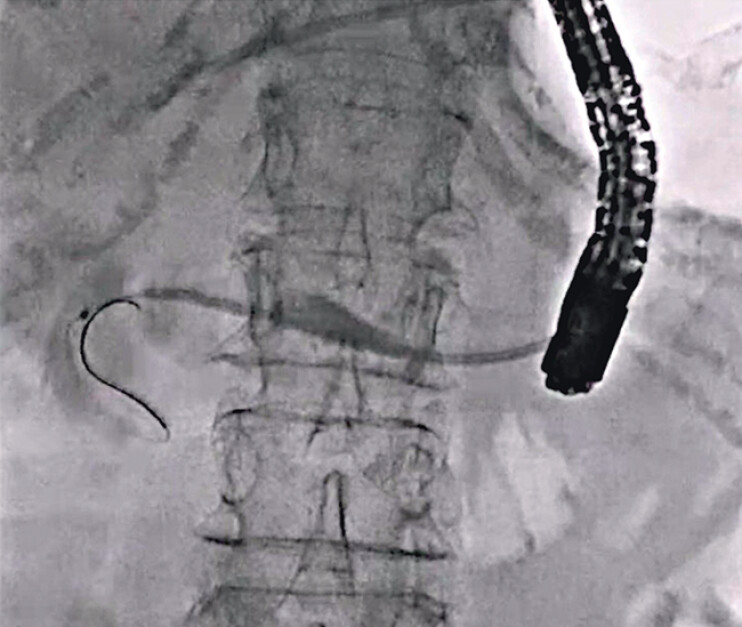
The contrast medium flowing into the intestine through the stricture is seen.

Antegrade radial laser ablation is performed.Video 1

In conclusion, antegrade RLA for a PJS might be safe and effective, although further evaluation is needed.

Endoscopy_UCTN_Code_TTT_1AR_2AI

## Notes

Endoscopy E-Videos https://eref.thieme.de/e-videos
*E-Videos* is an open access online section of the journal *Endoscopy* , reporting on interesting cases and new techniques in gastroenterological endoscopy. All papers include a high-quality video and are published with a Creative Commons CC-BY license. Endoscopy E-Videos qualify for HINARI discounts and waivers and eligibility is automatically checked during the submission process. We grant 100% waivers to articles whose corresponding authors are based in Group A countries and 50% waivers to those who are based in Group B countries as classified by Research4Life (see: https://www.research4life.org/access/eligibility/ ). This section has its own submission website at https://mc.manuscriptcentral.com/e-videos .
